# Enhanced treatment of dispersed dye-production wastewater by self-assembled organobentonite in a one-step process with poly-aluminium chloride

**DOI:** 10.1038/s41598-017-07333-2

**Published:** 2017-07-28

**Authors:** Yao Liu, Lizhong Zhu

**Affiliations:** 1 Department of Environmental Science, Zhejiang University, Hangzhou, Zhejiang 310058 China; 2 Zhejiang Provincial Key Laboratory of Organic Pollution Process and Control, Zhejiang University, Hangzhou, 310058 China

## Abstract

Organobentonite has been successfully applied in industrial wastewater treatment. However, the solid-liquid separation in wastewater treatment still needs improvement. This study presents an enhanced approach with high removal efficiency and short separation time for dispersed dye-production wastewater using self-assembled organobentonite in a one-step process with poly-aluminium chloride (PAC). The enhanced effects of PAC on wastewater treatment by organobentonite were comprehensively evaluated. Following the primary decontamination by the self-assembled organobentonite, the removal efficiency for dispersed dye-production wastewater was strengthened with PAC coagulation. The removal rates of TOC and organic pollutants were 55.0% and 63.5%, respectively, with the PAC-enhanced approach and were 1.3- and 1.6-fold higher, respectively, than those with the self-assembled organobentonite approach. The combination of PAC with self-assembled organobentonite was able to break the stability of the organobentonite suspension and enlarge the floc size, and thus reduce the solid-liquid separation time from 30 min to 10 min. Additionally, this enhanced approach could improve the biodegradability of wastewater with the BOD_5_/COD_Cr_ ratio increasing from 0.22 to 0.39, which was 4.1-fold higher than that of only organobentonite in a one-step process. Therefore, the PAC-enhanced approach could be a promising technology for wastewater pretreatment in practical industrial applications.

## Introduction

Dye-production wastewaters, generated during the manufacturing of dyes, have heavy colour, rich organic content, and serious toxicity. This type of wastewater contains the mixture of colourants and various aromatic organics, such as anilines, nitrobenzenes, and phenols, which may cause numerous hazardous water quality effects, including aesthetic problems, enhanced concentrations of organic compounds and toxicity to aquatic organisms^1, 2^. The biological treatment process is widely used in industrial wastewater treatment due to its feasibility and low cost^3, 4^. However, its effectiveness may be limited by the presence of recalcitrant and/or toxic organic compounds in dye-production wastewater. To ensure the efficiency and stability of biodegradation, a pretreatment procedure can be indispensable as it can reduce the refractory organics in the effluents and lower the toxicity.

Organobentonite is produced by exchanging inorganic cations in the interlayer of bentonite with organic cations and is increasingly attracting attention as a sorbent for its rich reserve and high sorption capacity. The one-step process of organobentonite is a mature approach for refractory organic wastewaters, and it has been successfully applied in the pretreatment of coking wastewater^5^. Traditional applications of organobentonite in wastewater treatment usually include two individual steps: a complicated organic modification of bentonite and the sorption of organic pollutants by synthesized organobentonite. The one-step process, including the synthesis of organobentonite and sorption of organic pollutants, was originally proposed to simplify the process of wastewater treatment by organobentonite^6^. In the process, bentonite powders and cationic surfactants were well dispersed and formed a stable suspension, and then organic pollutants were removed by self-assembled organobentonite through partitioning and adsorption. This process is considered to be a suitable pretreatment for dispersed dye-production wastewater due to its high removal capacities for organic pollutants, especially for those refractory hydrophobic pollutants^7–9^. It can remove cationic surfactant and organic pollutants simultaneously from the wastewater^10^, which could allow it to achieve similar or higher efficiency and lower cost than the traditional two-step process^11, 12^.

However, due to the high swelling ratio and tendency to remain suspended in water, the bentonite powder was found to be difficult to separate from treated wastewater by settlement^13, 14^. One of the most significant limiting factors of bentonite in industrial application has been the difficulty of separating and handling it^15, 16^. The precipitative properties of organobentonite, though they have been improved to some extent by organic modifications^17^, still need improvement for separation and recycling in industrial wastewater treatment^18^. In addition, the sorption capabilities of organobentonite have been reported to be correlated with the octanol-water partition coefficients of organic compounds^19^. Consequently, it has exhibited poor removal of hydrophilic aromatic compounds^20^, resulting in low removals for the total organic pollutants in highly concentrated wastewater.

Coagulants are widely used in the pretreatment system to destabilize and aggregate the suspended particles or precipitates and adsorb dissolved contaminants from wastewater. Chemical coagulation with inorganic coagulants such as poly-aluminium chloride (PAC), has been successfully applied in industrial wastewater treatment. Studies have also shown that clay can be used as a coagulation crystal nucleus to induce the formation of alum floc, which has also contributed to the enhanced removal of organic pollutants from wastewater^21, 22^. Therefore, the use of coagulation in the combination with organobentonite would promote the separation of organobentonite and enhance the removal efficiency, leading to significant reductions in both precipitation time and organic content.

In this study, an approach combining self-assembled organobentonite with PAC was developed for treating industrial dispersed dye-production wastewater. The optimal chemical dosages of the various system components were determined. The enhancement effect of PAC on the organobentonite method was evaluated through a series of analyses including the removal rates of colour, total organic carbon (TOC) and organic contaminants, the precipitation performance of bentonite, and the biodegradability of wastewater. The precipitation performance of bentonite and organobentonite with sorbed organics was evaluated in terms of the residual turbidity of the supernatant as a function of precipitation time. The zeta potential values of organobentonite with sorbed organics, as well as the floc size, were further investigated. This study aimed to provide an enhanced approach for dispersed dye-production wastewater treatment and technical support to its application.

## Results and Discussion

### Dosage effects of bentonite and cetyltrimethylammonium bromide on the removal of selected pollutants

**Figure 1:**
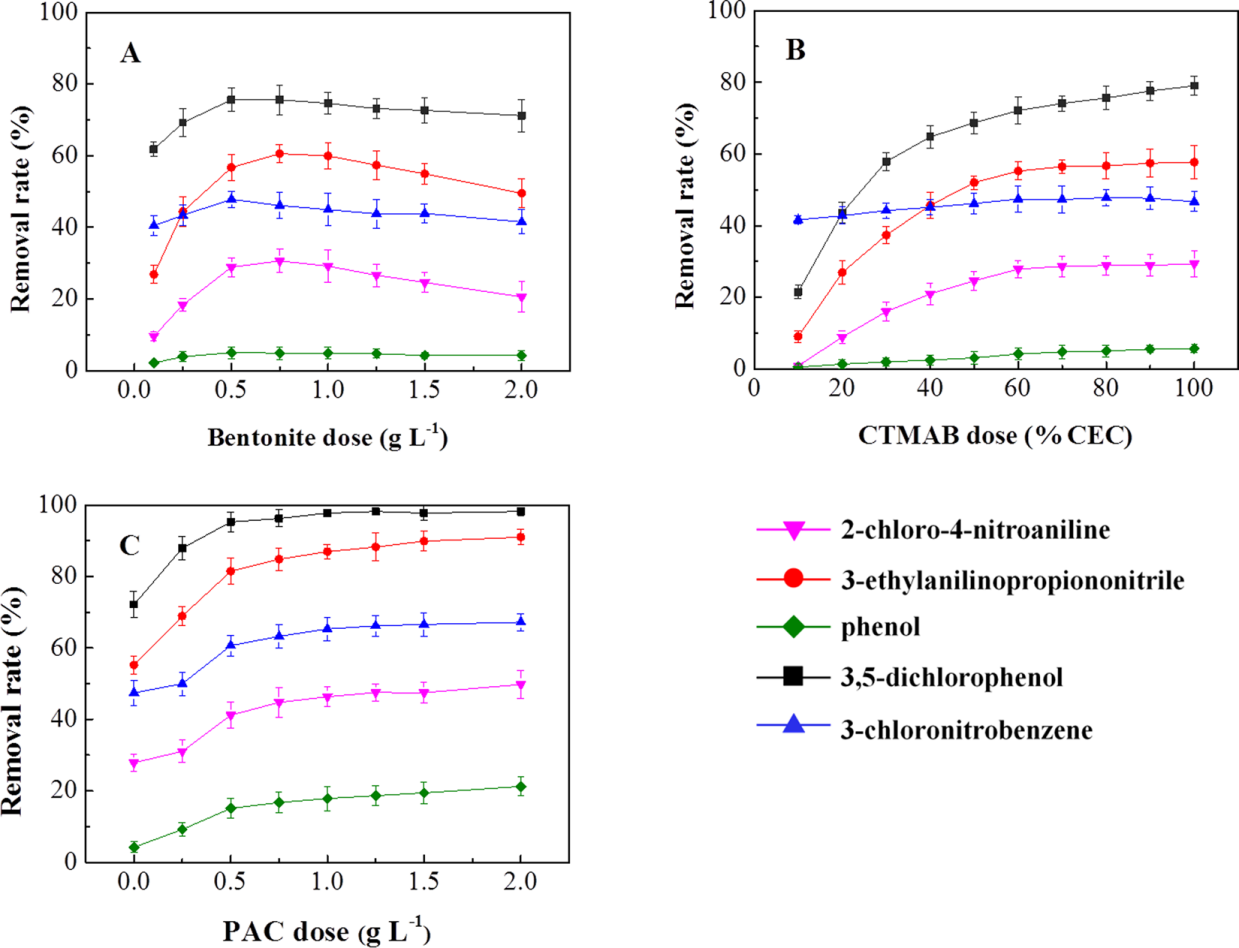
Dose effects of bentonite, CTMAB and PAC on the removal rates of selected organic pollutants. (**A**) CTMAB dose is fixed at 100 mg L^−1^, (**B**) bentonite dose is fixed at 0.5 g L^−1^, (**C**) bentonite and CTMAB dose is fixed at 0.5 g L^−1^ and 60% cation exchange capacity, respectively. Error bars represent standard deviations (*n* = 3).

The sorption efficiency of the bentonite was dependent on the types of organic pollutants. When bentonite dosage was increased from 0.1 g L^−1^ to 2.0 g L^−1^ and cetyltrimethylammonium bromide (CTMAB) dosage was kept at 100 mg L^−1^, the CTMAB loading per unit bentonite of the synthesized organobentonite was lowered from 400% cation exchange capacity (CEC) to 20% CEC (Fig. 1A). Higher dosages of bentonite would increase the physical adsorption ability but would lower the partition effect of the surfactant layer in the organobentonite^9, 23^. For the anilines, the breaking point of the removal effect was found to be at the bentonite dosage of 0.5 g L^−1^. The removal rates of 3-chloronitrobenzene and phenols were essentially unchanged throughout the whole process, indicating that bentonites were not very effective in removing those classes of pollutants, which might be caused by the hydrophobicity and low organic carbon content of the bentonite^24, 25^. Because of these experiments, the bentonite dosage was selected at 0.5 g L^−1^ for the best removal of five kinds of representative pollutants.

Surfactants played a key role in the performance of organobentonite in wastewater treatment. With the bentonite dosage held constant at 0.5 g L^−1^, the removal rates of organics increased as the CTMAB dosage increased from 10% CEC to 60% CEC (Fig. 1B). Within the experimental range, almost all (>95%) CTMA^+^ was removed by bentonite in the one-step process. With the same CTMAB dosage increase, the removal rates of 2-chloro-4-nitroaniline and 3,5-dichlorophenol increased from 0.9% to 27.9% and from 21.5% to 72.1%, respectively. As the dosage increased, the interlayer packing densities increased, the interlayer space expanded, and the interaction with the alkyl chain was stronger for CTMA^+^ in the bentonite to form a more powerful organic partitioning phase^26^. The removal rates increased slightly when the CTMAB dosage was more than 60% CEC, but in consideration of the removal efficiency and cost, the optimal experimental dosage was selected as 0.5 g L^−1^ of bentonite and 60% CEC of CTMAB for the one-step process.

For a further investigation of the structure of the adsorbate layer, the XRD patterns were employed for the investigation of the interlayer structures of bentonite, traditional synthesized organobentonite in the two-step process and self-assembled organobentonite in the one-step process. The XRD results showed that the basal spacing of bentonite had swelled from 1.49 nm to 2.06 nm after it had sorbed CTMA^+^ of 60% CEC in the one-step process, which had only a negligible difference from traditional synthesized organobentonite (Fig. S1). The basal space of traditional synthesized organobentonite in the two-step process was 2.09 nm. Similar increments for the interlayer spacing were also observed in a previous study^6^. The increase of the interlayer spacing suggested that the CTMA^+^ had intercalated into the layers and formed an extended structure with a tilt angle, which would also suggest the self-assembly of organobentonite in the one-step process.

### Removal efficiency for dispersed dye-production wastewater

Based on the selected optimal dosage, the decolourization and TOC removal of organobentonite in the one-step process was investigated for dispersed dye-production wastewater. The TOC of wastewater (shown in Fig. S2), treated by the one-step process, decreased from 3263.2 to 1722.2 mg L^−1^ for DR 145 and from 14210.0 to 9836.8 mg L^−1^ for DR 167 (DR 145 and DR 167 stand for the wastewater generated during the manufacturing process of C.I. Disperse Red 145 (heterocyclic azo dye) and C.I. Disperse Red 167 (monoazo dye), respectively). In addition, the colour of wastewater dramatically decreased from 25000 to 1350 and 16000 to 3277 for the two respective wastewaters. The one-step process of organobentonite achieved good decolourization performance of dispersed dye-production wastewater, which suggests that it has promising prospects for industrial application. However, the TOC removal in the one-step process was relatively low and left much room for improvement.

Co-precipitation, which is often used as a method to improve the efficiency of the pretreatment process of wastewater, was applied in the combination of the one-step process. With the bentonite and CTMAB dosages fixed at 0.5 g L^−1^ and 60% CEC, respectively, the PAC dosage was evaluated from 0 to 2.0 g L^−1^ (Fig. 1C). When the dosage was 0.5 g L^−1^, the removal rates of the five types of organic pollutants were 15.1%, 41.2%, 60.7%, 81.4% and 95.3%, respectively, much higher than the 4.2%, 27.9%, 47.4%, 55.2%, and 72.1% achieved without PAC treatment. It could be that the PAC hydrolysis product was capable of adsorption bridging with bentonite, and of sorbing organic matter^27, 28^. The removal rate kept increasing with PAC dosage until the dosage was higher than 0.5 g L^−1^. Thus, the optimal experimental dosage was selected as 0.5 g L^−1^ of bentonite, 60% CEC of CTMAB and 0.5 g L^−1^ of PAC for the one-step process.

**Figure 2:**
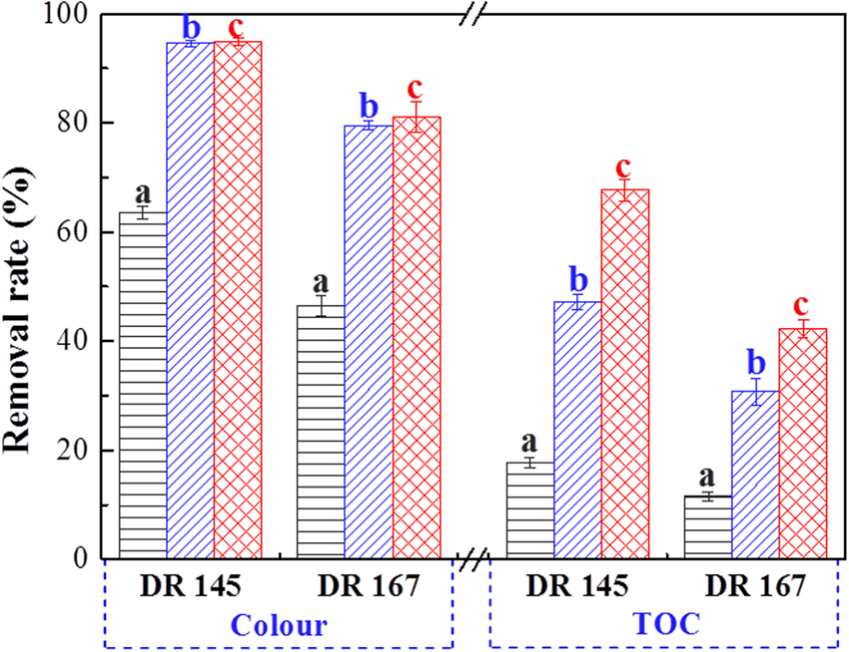
Decolourization and TOC removal of dispersed dye-production wastewater by (**a**) PAC coagulation, (**b**) the one-step process of organobentonite and (**c**) the one-step process with PAC. Bentonite dose: 0.5 g L^−1^, CTMAB dose: 60% CEC, PAC dose: 0.5 g L^−1^. Error bars represent standard deviations (*n* = 3).

When combined with PAC, the one-step process was observed to be effective in both decolourization and TOC removal for dispersed dye-production wastewater (Fig. 2). The wastewater samples in this research were actual industrial dye manufacturing effluents with very high colour, obtained directly from the factory. The decolourization rate of PAC coagulation was 63.6% for DR 145 and 46.5% for DR 167. With the selected optimal dosage applied in the one-step process with PAC, the decolourization rate was 94.8% and 81.1% for DR 145 and DR 167, respectively. Various types of wastewater pretreatment technologies, such as coagulation and Fenton oxidation, were previously reported to be effective in colour removal (from 72% to 94%) for simulated disperse dye solutions^29^. When applied to actual industrial dye-production wastewater, however, the colour removal was lower^30, 31^ (from 71% to 74%). The one-step process with PAC was very effective in colour removal for actual dispersed dye-production wastewater. Furthermore, the TOC removal achieved by this approach was 67.7% and 42.3% for DR 145 and DR 167, respectively, which is similar to the widely applied treatments of industrial wastewater^32, 33^. The enhancement by PAC coagulation was mainly embodied in TOC removal, which was 16.0% higher than that without PAC and was 3.7-fold higher than PAC alone. The results proved the one-step process with PAC to be an effective approach for the treatment of dispersed dye-production wastewater.

### PAC enhancements of self-assembled organobentonite for wastewater treatment

#### Removal efficiency of organic pollutants

**Figure 3:**
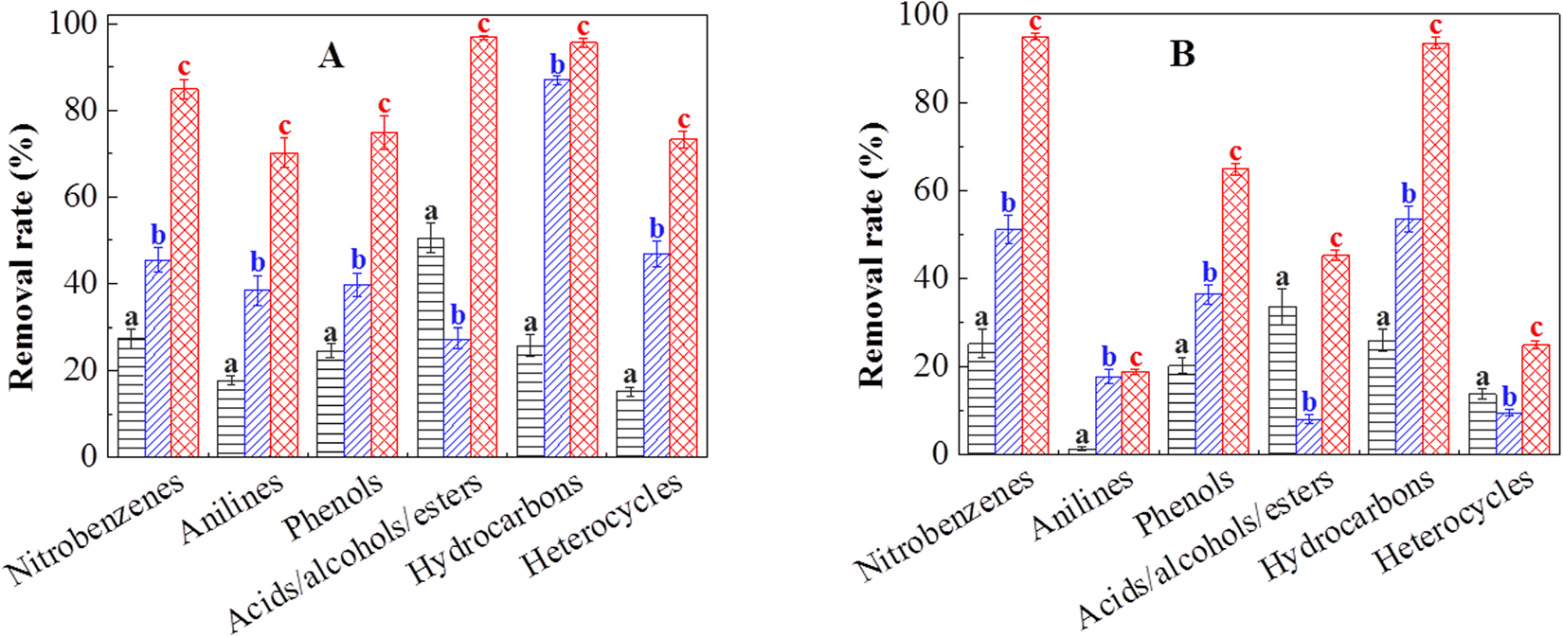
Removal rate of organic contaminants by (a) PAC coagulation, (b) the one-step process of organobentonite and (c) the one-step process of organobentonite with PAC for (**A**) DR 145 and (**B**) DR 167. Bentonite dose: 0.5 g L^−1^, CTMAB dose: 60% CEC, PAC dose: 0.5 g L^−1^. Error bars represent standard deviations (*n* = 3).

The organic composition of wastewater was characterized before and after the one-step process with or without PAC treatment. As shown in Fig. 3, following the primary decontamination by self-assembled organobentonite in the one-step process, removal of organic pollutants was enhanced by PAC coagulation. In the one-step process, the six categories of pollutants showed average removal rates of 70.2%, 48.2%, 17.7%, 38.1%, 28.1% and 28.0% for hydrocarbons, nitrobenzenes, acids/alcohols/esters, phenols, heterocycles, and anilines, respectively. After adding PAC, those removal rates increased to 94.5%, 89.9%, 71.0%, 69.8%, 49.1% and 44.5%, respectively, which represented a substantial improvement in decontamination. The removal rates with PAC alone for the six categories of pollutants were lower than those of the one-step process (shown in Fig. 3). The combination of PAC and organobentonite achieved a synergistic removal of nitrobenzenes and anilines. In the one-step process, the total concentrations of organic compounds in DR 145 and DR 167 reduced by 48.3% and 30.0%, respectively. The total removal of organic pollutants by PAC coagulation was 18.3% for DR 145 and 12.1% for DR 167, while in the one-step process with PAC, the total decontamination rates were 78.2% and 48.7% for dispersed dye-production wastewater (DR 145 and DR 167, respectively). The results indicate that PAC coagulation can improve the total decontamination of a one-step process for dispersed dye-production wastewater.

**Figure 4:**
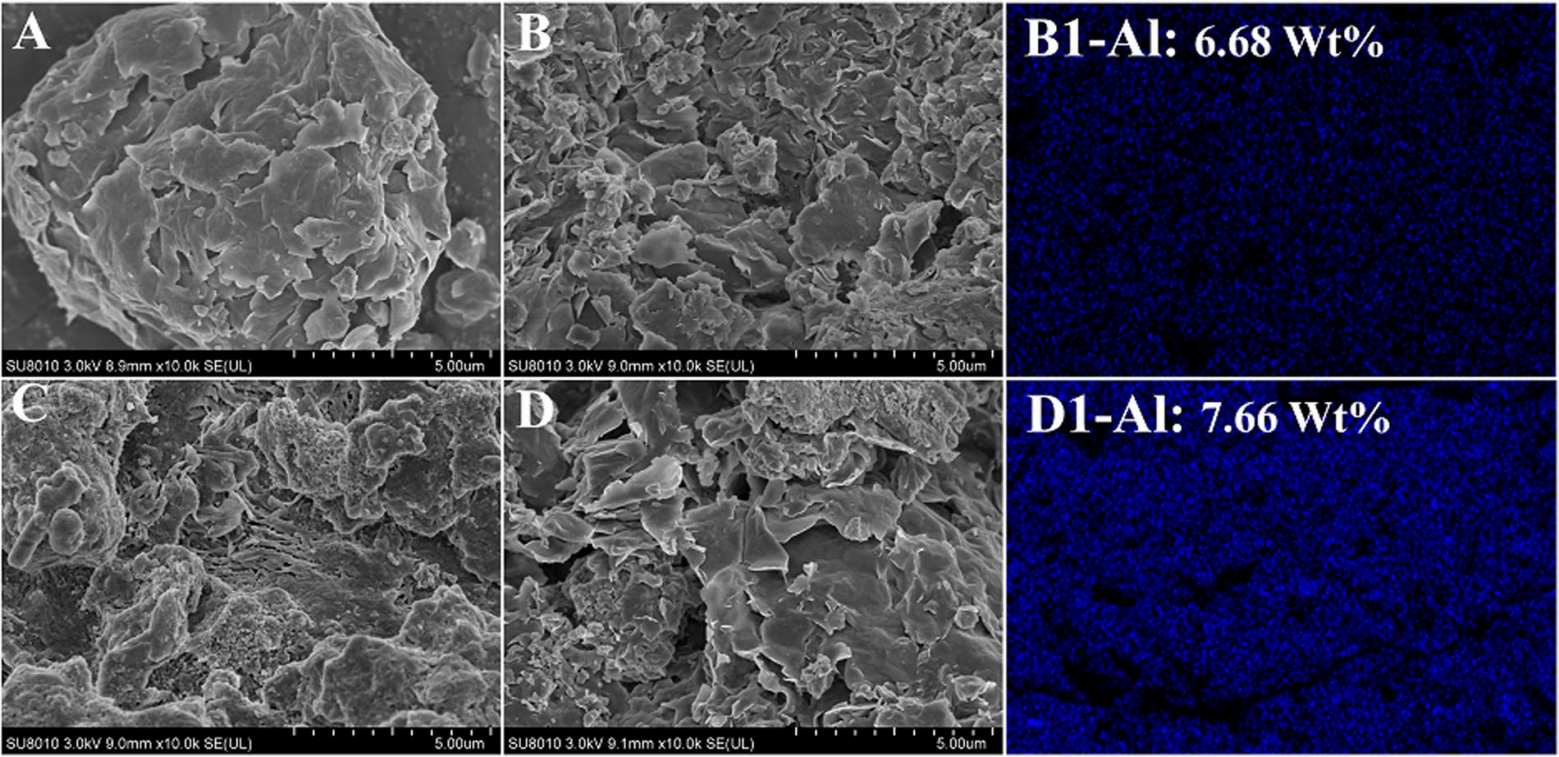
Scanning election micrographs and element surface distribution graphs of Al: (**A**) bentonite, (**B**) and (B1) organobentonite in the one-step process, (**C**) flocs in PAC coagulation, (**D**) and (D1) organobentonite in the one-step process with PAC.

The scanning electron microscope (SEM) micrographs provided a glance at the surface structure of the bentonite and organobentonite (shown in Fig. 4). The surface of natural bentonite (Fig. 4A) was tightly packed with a typical flat flaky texture, as reported previously^34^. The self-assembled organobentonite (Fig. 4B) in the one-step process exhibited a curly lamellar structure with a rough surface and high porosity, which could lend it better sorption performance due to the larger surface area. In the one-step process with PAC, the sorbed organobentonite transformed to coarse and aggregated morphology (Fig. 4D). There were some amorphous particles in both organobentonite agglomerations (Fig. 4D) and the flocs formed by PAC coagulation alone (Fig. 4C). The particles have a diameter of approximately 100 nm and were identified as aluminium by the energy dispersive spectroscopic analysis. The results indicated that the PAC hydrolysis product was capable of adsorption bridging with sorbed organobentonite, forming porous agglomerations to improve the removal efficiency of both organic pollutants and sorbed organobentonite.

#### Precipitation performance of organobentonite with sorbed organics

**Figure 5:**
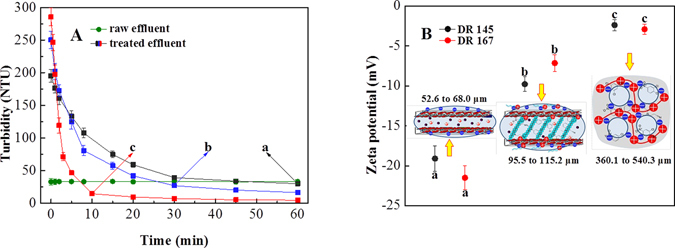
The variations in organobentonite settling properties: (**A**) residual turbidities and (**B**) zeta potential of (a) bentonite suspended effluent, organobentonite suspended effluent in (b) the one-step process and (c) the one-step process with PAC. Bentonite dose: 0.5 g L^−1^, CTMAB dose: 60% CEC, PAC dose: 0.5 g L^−1^. Error bars represent standard deviations (*n* = 3).

In the precipitation performance test, it was observed that the organobentonite with sorbed organics began to agglomerate and rapidly grew into thick flocs after the addition of PAC. It was found in previous studies that the major drawback of the one-step process was that it could be very time-consuming^18, 35^; in our research, the solid-liquid stratification for the one-step process was observed after 30 min of the precipitation, while the sorbed organobentonite settled in less than 10 min with the addition of PAC (Fig. 5A). The time for the settling process was shortened in this combined treatment. It was also observed that the sludge volume of sorbed organobentonite in the one-step process was reduced after the addition of PAC. The precipitate from the combined process was much easier to recycle and reuse in the industrial applications compared to traditional treatments^36^. The results showed that PAC could enhance the precipitation performance of self-assembled organobentonite in the one-step process, and thus achieved rapid separation and better recycling ability.

The zeta potential values (shown in Fig. 5B), which reflected the stability of the clay solution, were the key to understanding the outstanding performance of the PAC coagulation after the one-step process. The initial bentonite suspension had a −20.3-mV zeta potential value in dispersed dye-production wastewater and thus formed a negative stable system. The electronegativity of the suspension decreased to −8.5 mV after the addition of the cationic surfactant (CTMAB). CTMA^+^ could adsorb to the bentonite surface through electrostatic attraction and neutralize its charge^33, 37^, leading to a less negative and more flocculated system. After the PAC coagulation treatment, the zeta potential of organobentonite with sorbed organics further decreased to −2.6 mV. The cationic hydrolysis products of PAC could break the stability of the organobentonite suspension by neutralizing the negative charge of bentonite^38^, which would promote the coagulation of unstable colloids. In the one-step process with PAC, the floc size of bentonite particles increased from 60.3 μm to 105.4 μm with the presence of CTMAB and further increased to 450.2 μm with the addition of PAC (Fig. 5B). These results indicated that the addition of PAC could break the stability of the organobentonite suspension and increase the floc size, which would accelerate the solid-liquid separation.

#### Biodegradability of wastewater

**Figure 6:**
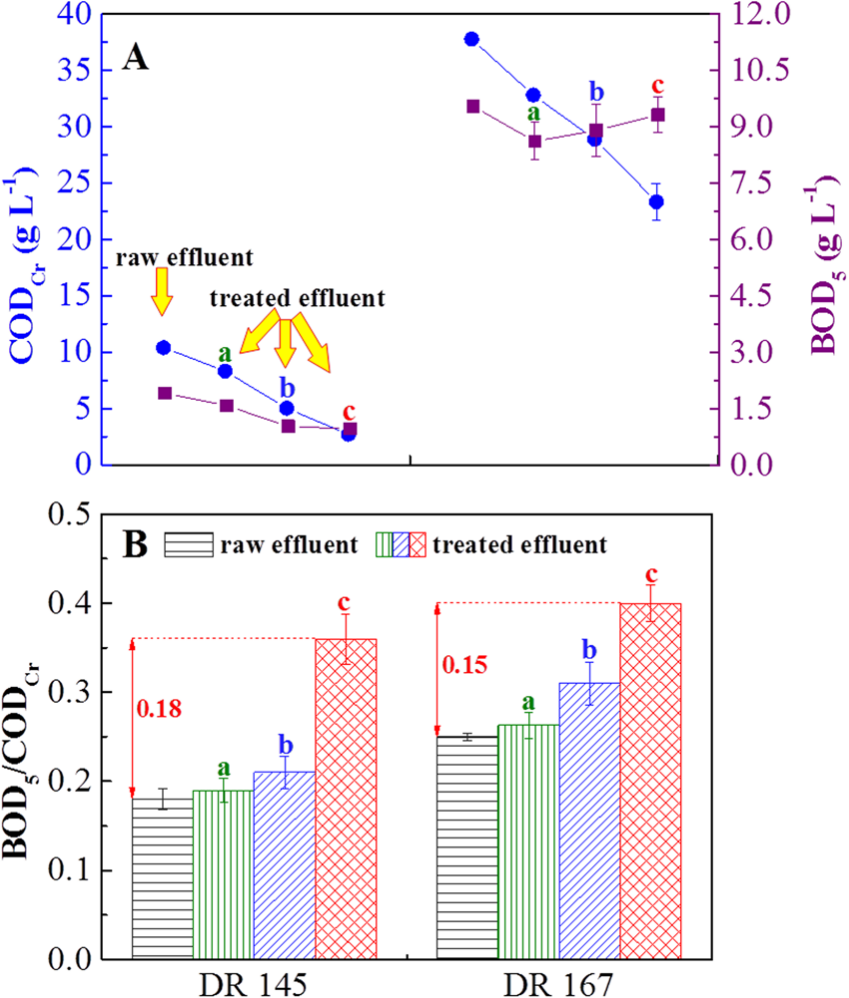
Effect of the treatment using (a) PAC coagulation, (b) the one-step process of organobentonite and (c) the one-step process of organobentonite with PAC on (**A**) COD_Cr_, BOD_5_ and (**B**) BOD_5_/COD_Cr_ ratio of dispersed dye-production wastewater. Bentonite dose: 0.5 g L^−1^, CTMAB dose: 60% CEC, PAC dose: 0.5 g L^−1^. Error bars represent standard deviations (*n* = 3).

For investigating the biodegradability of the wastewater treated by the physicochemical treatment methods, the COD_Cr_ and 5 days biochemical oxygen demand (BOD_5_) were determined using wastewater samples. The COD_Cr_ values of wastewater (Shown in Fig. 6A) treated by a one-step process with PAC decreased from 10.39 to 2.72 and from 37.74 to 25.08 g L^−1^ for DR 145 and DR 167, respectively. However, the reduction in BOD_5_ values was 1.04 and 0.61 g L^−1^ for the two respective dispersed dye-production wastewater samples, which was less significant. The COD_Cr_ removal rate of the treatments ranked as follows: one-step process with PAC > one-step process > PAC coagulation, while the BOD_5_ removal ranked in an opposite order. These results demonstrated that it was predominantly the refractory organics, rather than the biodegradable compounds, which were removed by the one-step process with PAC.

The biodegradability of raw and treated dispersed dye-production wastewater, evaluated through a calculation of the BOD_5_/COD_Cr_ ratio, is shown in Fig. 6B. The BOD_5_/COD_Cr_ ratios of raw wastewater were 0.18 and 0.25 for DR 145 and DR 167, respectively. The biodegradability of effluents treated by PAC coagulation showed little difference from the raw wastewaters. This indicated that using the one-step process with PAC method led to noticeable improvement in the biodegradability index (BOD_5_/COD_Cr_) for dispersed dye-production wastewaters. The index of treated effluent increased by 0.17 (from 0.18 to 0.36, and from 0.25 to 0.40 for DR 145 and DR 167, respectively) on average with the one-step process with PAC, which was 4.1-fold higher than that without PAC. Therefore, wastewater became more easily degraded by a biological reaction after treatment with the one-step process with PAC.

**Figure 7:**
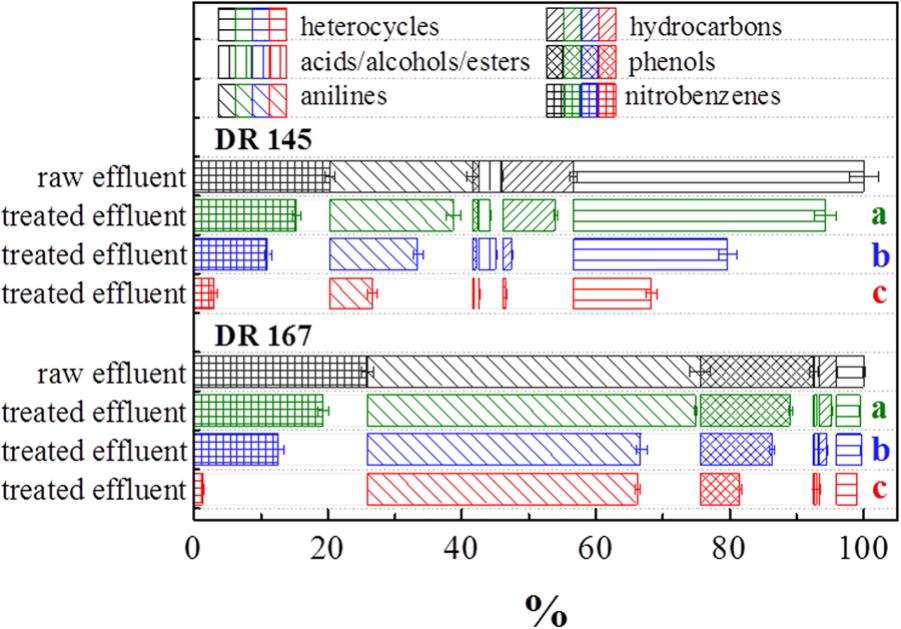
Organic composition of dispersed dye-production wastewater treated by (**a**) PAC coagulation, (**b**) the one-step process of organobentonite and (**c**) the one-step process with PAC. Bentonite dose: 0.5 g L^−1^, CTMAB dose: 60% CEC, PAC dose: 0.5 g L^−1^. Error bars represent standard deviations (*n* = 3).

The lowered organic content in dispersed dye-production wastewater is the main reason for the improvement in biodegradability. As shown in Fig. 7, the organic content of the treated effluents, especially those treated by organobentonite in one-step process with PAC, was effectively removed. Moreover, the organic composition in the wastewater was altered by the treatments. Anilines and nitrobenzenes were found to be the main organic constituents in dispersed dye-production wastewater. The nitrobenzenes/anilines ratio of DR 145 and DR 167, which could be considered as a marker of the organic composition, was reduced by the one-step process with PAC, from 95.1% to 48.4% and 51.9% to 3.2%, respectively. Therefore, the one-step process with PAC was proven to have the ability to reduce the organic content and alter the composition of dispersed dye-production wastewater. Nitrobenzenes were reported to be hardly removed by aerobic degradation which was restricted by the electronegative nitro group^39^. It was reported that anilines had lower toxicity than nitrobenzenes and were easily mineralized by aerobic degradation^40, 41^. As such, the alteration of the organic composition could also enhance the biodegradation ability of dispersed dye-production wastewater.

**Figure 8:**
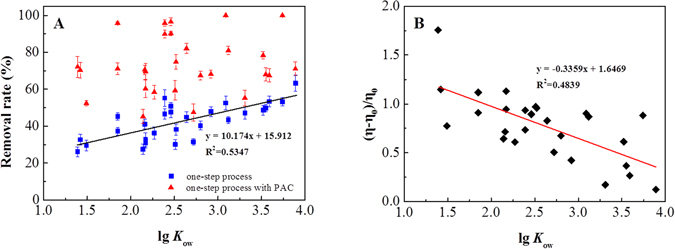
Relation between removal rate of the processes. (**A**) PAC enhancement, (**B**) lg*K*_ow_ of aromatic organics. Bentonite dose: 0.5 g L^−1^, CTMAB dose: 60% CEC, PAC dose: 0.5 g L^−1^. Error bars represent standard deviations (n = 3).

The removal efficiency of organic pollutants by organobentonite was generally correlated with the hydrophobicity (using lg*K*_ow_ as a factor), which did not apply to the one-step process with PAC. As shown in Fig. 8A, the removal rate of aromatic pollutants in the one-step process was positively correlated with the lg*K*_ow_ of compounds (*r* ^2^ = 0.535, *p* < 0.01). If the hydrophobicity of the aromatic pollutants strengthened, the adsorption effect got stronger. Similar correlations between the hydrophobicity and the adsorption effect on organobentonite were observed in previous research^5^. However, the lg*K*_ow_ and removal rate showed no significant correlation (*p* > 0.05) in the one-step process with PAC. The PAC is capable of non-selective adsorption and co-coagulation of organic pollutants^42^, which might be the reason for the lack of correlation between the lg*K*_ow_ and the removal of organic pollutants. The strengthening effect of PAC (shown in Fig. 8B) was negatively correlated with the lg*K*_ow_ of compounds (*r* ^2^ = 0.484, *p* < 0.01). In addition to the high efficiency in removing hydrophobic pollutants, the negative correlation in the one-step process with PAC represented a removal potential for hydrophilic organics as well. Therefore, the use of PAC in the combination with organobentonite would compensate for the weakness of organobentonite and thus promote its practical industrial application.

In summary, adding PAC could enhance the decontamination of self-assembled organobentonite and shorten the solid-liquid separation time in the treatment of dispersed dye-production wastewater. The PAC-enhanced approach was effective in overall decontamination and in the prior removal of recalcitrant organics, and thus improved the biodegradability of wastewater. Therefore, it could be a promising technology for wastewater pretreatment in practical industrial applications.

## Methods

### Materials

The sodium bentonite raw material was purchased from Chengbei Co., Ltd. (Zhejiang, China). The cation exchange capacity (CEC) was 0.696 mmol g^−1^ bentonite. Analytical-grade cetyltrimethylammonium bromide (CTMAB) was selected to modify the bentonite. 2-chloro-4-nitroaniline, 3-ethylanilinopropiononitrile, phenol, 3,5-dichlorophenol, and 3-chloronitrobenzene were of analytical grade. Analytical-grade PAC (containing 27% as Al_2_O_3_) with a basicity of 70–75% was used as the coagulant and was purchased from Dingshengxin Chemical Co., Ltd. (Tianjin, China). Other chemicals used in this study were purchased from the Aladdin Chemical Reagent Co., Ltd., China.

The dispersed dye-production wastewater, with colour and TOC from 16000 to 25000 and from 3263.2 to 14210.0 mg L^−1^, respectively, was collected from a dyestuff chemical factory in Zhejiang, China. The detailed characteristics of the dispersed dye-production wastewater are provided in Table 1. The molecular structures of the disperse dyes and their syntheses are shown in Fig. S3.

**Table 1:** Characteristics of dispersed dye-production wastewater.

Wastewater sample parameters	DR 145^a^	DR 167^a^
Ph	1.24	2.02
Colour^b^	25000 ± 1000	16000 ± 2375
TOC^b^ (mg L^−1^)	3263.2 ± 87.2	14210.0 ± 418.0
COD_Cr_^b^ (mg L^−1^)	10391.0 ± 157.4	37736.4 ± 246.0
BOD_5_^b^ (mg L^−1^)	1916.7 ± 121.6	9538.9 ± 125.1
BOD_5_/COD_Cr_^b^	0.18 ± 0.012	0.25 ± 0.004

^a^DR 145 and DR 167 are the abbreviations of wastewater generated from the manufacturing processes of C.I. Disperse Red 145 and C.I. Disperse Red 167, respectively.

^b^Mean ± standard deviation (*n* = 3).

### Sorption-coagulation experiment

Representative aromatic organics in dispersed dye-production wastewater, such as 2-chloro-4-nitroaniline, 3-ethylanilinopropiononitrile, phenol, 3,5-dichlorophenol, and 3-chloronitrobenzene, were used to investigate the optimal dosages of bentonite, CTMAB, and PAC^1, 43^. The detailed characteristics of the selected pollutants are shown in the Supporting Information (Table S1). The chosen concentrations were 100 mg L^−1^ for hydrophilic organic compounds and 10 mg L^−1^ for hydrophobic organic compounds with a comparative lower water solubility^44^. In the optimization process, the selected amount of bentonite and CTMAB was added into a 22-mL sampling tube. Then, 20 mL of a solution of the selected organic compound at a certain concentration was added separately, and the sampling tube was sealed and shaken at 25 °C and 200 rpm. For the coagulation treatment, the PAC was added after the sorption equilibrium had been reached, then the sample was stirred at 25 °C and 50 rpm for 30 min. All of the samples were centrifuged at 3000 rpm for 20 min. The supernatant was extracted by n-hexane and used for further analysis by GC-MS.

For evaluating the removal efficiency between two physicochemical treatments, the combined one-step process with PAC coagulation was performed as a two-step treatment whereby sorption was followed by coagulation. Bentonite and CTMAB (100% CEC indicated that the CTMAB dosage corresponds to 100% of bentonite CEC satisfied by CTMAB) were premixed and added to 200 mL dispersed dye-production wastewater in a 250-mL container. The sorption process was conducted under comparatively rapid agitation at 200 rpm for 30 min. PAC was added when the sorption equilibrium had been achieved. The suspension was stirred at a lower speed of 50 rpm for 10 min. After a static settlement for 10 min, the supernatant was separated for further clean-up and extraction.

A settlement experiment was conducted in a 3-L container. A certain amount of bentonite and CTMAB were added to 2 L dispersed dye-production wastewater, and the mixture was stirred at 200 rpm for 30 min. PAC was added when the sorption equilibrium had been achieved. Then, the suspension was stirred at 50 rpm for 10 min. The residual turbidity of the supernatant was measured continuously throughout a 60-min settling period.

### Chemical analysis and characterization

Analysis of samples for colour, BOD_5_, COD_Cr_ and turbidity were determined according to China National Standard Methods (GB 11903-89, HJ 505-2009 and GB 11914-89 and GB 13200-91, respectively). The TOC in the raw and treated wastewater was measured with a TOC analyser (TOC-VCPH, Shimadzu). Zeta potentials of organobentonite with sorbed organics and floc size were measured with a Zetasizer (Nano ZS90, Malvern) and a laser granulometer (Mastersizer 2000, Malvern), respectively. The surface morphologies and microscopic features of sorbed bentonite and organobentonite were determined using scanning electron microscopy (SEM, Hitachi SU8010) at an operating voltage of 3 kV. The powder samples were sputter-coated with a thin layer of gold and X-ray diffraction (XRD) patterns of the prepared samples were acquired with an X-ray diffractometer (XRD 6000, Shimadzu) using Cu Kα radiation (40 kV,30 mA). All XRD patterns were obtained from 3° to 20° with a scan speed of 4° min^−1^.

The raw and treated wastewater needed to be cleaned up by solid-phase extraction (SPE) before it could be analysed by GC-MS. Before the extraction process, the SPE C18 column was washed with 5 mL dichloromethane, followed by 5 mL methanol and finally by 5 mL deionized water. After a 20-fold dilution with deionized water, 200 mL raw and treated dispersed dye-production wastewater together with 20 mL methanol was percolated through the SPE-C18 column assisted by a vacuum pump. 5 mL deionized water was used to wash the column. The trapped organic pollutants were eluted with 7 mL dichloromethane (3:2:2, extracting 3 times). The solution was concentrated to near dryness with gentle nitrogen flow and then diluted to 1 mL with n-hexane for GC-MS analysis.

The quantitative analysis of target organic compounds in the wastewater was carried out by a gas chromatography (Agilent 7890B) equipped with 5977 A mass spectrometer operated in the electron impact (EI) mode. Aliquots of 1 µL were splitlessly injected into an HP-5 MS UI column (30 m, 0.25 mm i.d., 25 μm thick, Agilent Inc.). The temperature programme of the GC analysis was as follows^1^: the initial temperature at 45 °C, held for 2 min; increased by 15 °C min^−1^ to 150 °C, then held for 2 min; increased by 2 °C min^−1^ to 220 °C, then held for 2 min; increased by 30 °C min^−1^ to 300 °C; then held for 15 min. The injector was kept at 250 °C. The carrier gas, helium, constantly flowed at 1.0 mL min^−1^.

## Electronic supplementary material

Supporting Information
