# Supplementary material for: Enhanced treatment of dispersed dye-production wastewater by self-assembled organobentonite in a one-step process with poly-aluminium chloride
Source: Sci Rep. 2017 Jul 28;7:6843. doi: 10.1038/s41598-017-07333-2 (PMC5533790; doi:10.1038/s41598-017-07333-2)
Supplement: Supplementary file 1 — Supporting Information [file 41598_2017_7333_MOESM1_ESM.pdf]

Supporting Information

Enhanced treatment of dispersed dye-production wastewater by self-assembled organobentonite in a one-step process with poly-aluminium chloride

Yao Liu[a,b] and Lizhong Zhu[*, a, b]

[a]Department of Environmental Science, Zhejiang University, Hangzhou, Zhejiang 310058, China

[b]Zhejiang Provincial Key Laboratory of Organic Pollution Process and Control, Hangzhou, Zhejiang University 310058, China

[*]Corresponding author: Lizhong Zhu

Phone/Fax: +86 57188273733

E-mail: zlz@zju.edu.cn

Contents

Table S1 Physiochemical properties of the selected organic pollutants.

Figure S1 XRD patterns of bentonite, traditional synthesized organobentonite and self-assembled organobentonite in the one-step process.

Figure S2 Decolourization and TOC removal of dispersed dye-production wastewater by the one-step process of organobentonite.

Figure S3 Molecular structures and syntheses of disperse dyes.

Table S1 Physiochemical properties of the selected organic pollutants.

**Compound**	**Formula**	M_W	S_w **(ppm)**	**lg** K_{ow}	C_i **(ppm)**
2-chloro-4-nitroaniline	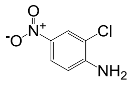	173	889	2.14	3.86[b]
3-ethylanilinopropiononitrile	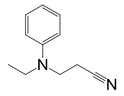	174	443	2.17	1.32[a]
phenol	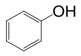	94	26160	1.46	0.13[a],4.96[b]
3,5-dichlorophenol	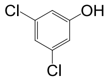	163	204	3.62	3.97[b]
3-chloronitrobenzene	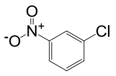	158	227	2.46	0.07[a],4.52[b]

[a] DR 145, [b] DR 167.

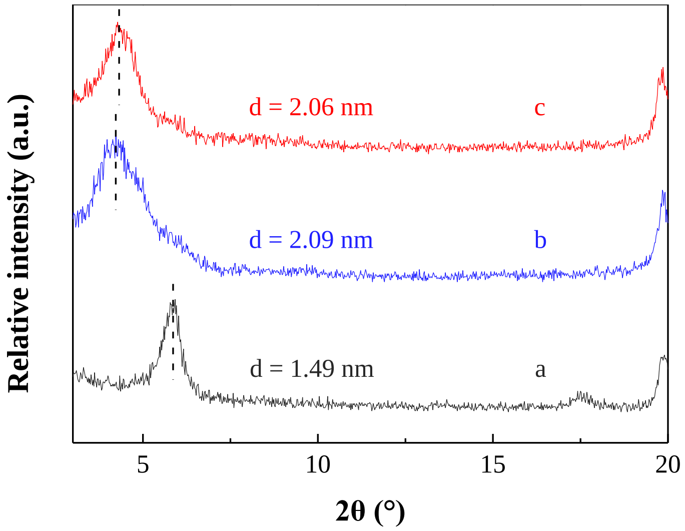

Figure S1 XRD patterns of bentonite (a), traditional synthesized organobentonite (b) and self-assembled organobentonite in the one-step process (c). Bentonite dose: 0.5 g L^{-1}, CTMAB dose: 60% CEC.

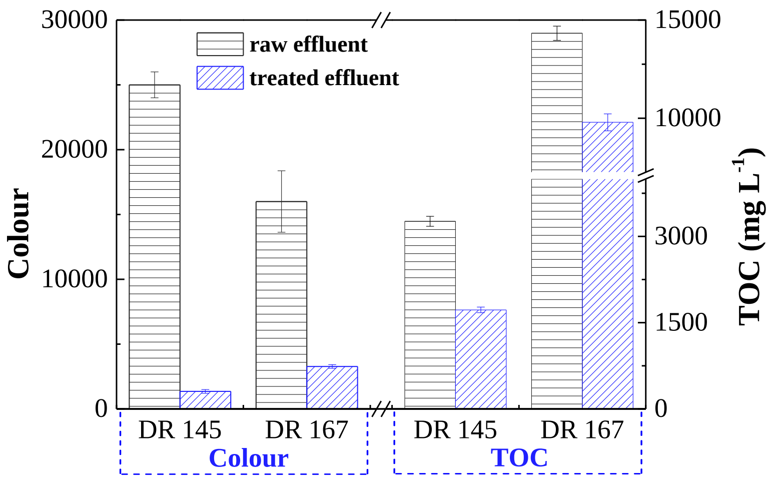

Figure S2 Decolourization and TOC removal of dispersed dye-production wastewater by the one-step process of organobentonite. Bentonite dose: 0.5 g L^{-1}, CTMAB dose: 60% CEC. Error bars represent standard deviations (n=3).

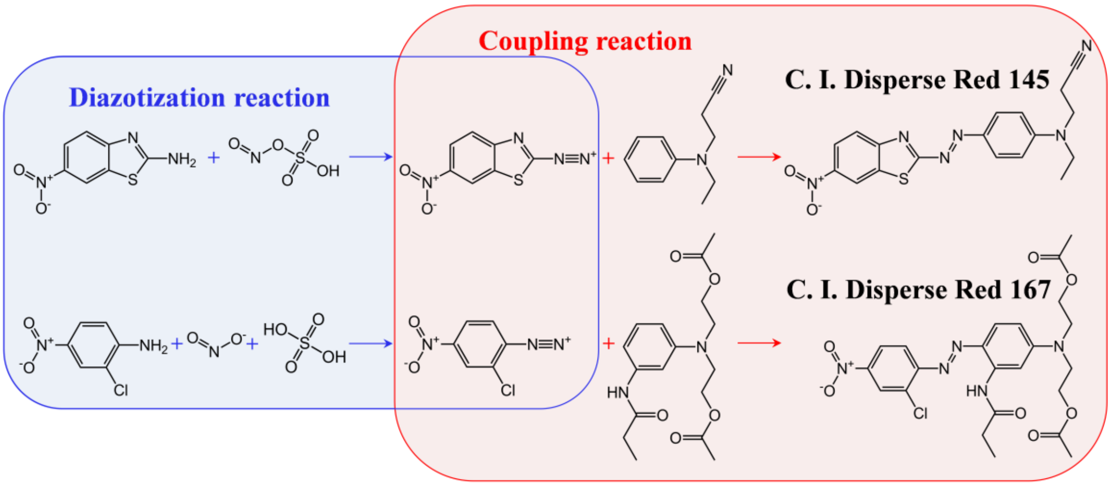

Figure S3 Molecular structures and syntheses of disperse dyes.
